# TL1A/DR3 Axis, A Key Target of TNF-a, Augments the Epithelial–Mesenchymal Transformation of Epithelial Cells in OVA-Induced Asthma

**DOI:** 10.3389/fimmu.2022.854995

**Published:** 2022-03-14

**Authors:** Dong Zhang, Hui Yang, Xue-Li Dong, Jin-Tao Zhang, Xiao-Fei Liu, Yun Pan, Jian Zhang, Jia-Wei Xu, Zi-Han Wang, Wen-Jing Cui, Liang Dong

**Affiliations:** ^1^ Department of Respiratory, Shandong Provincial Qianfoshan Hospital, Cheeloo College of Medicine, Shandong University, Jinan, China; ^2^ Department of Respiratory, The First Affiliated Hospital of Shandong First Medical University and Shandong Provincial Qianfoshan Hospital, Shandong Institute of Respiratory Diseases, Jinan, China

**Keywords:** TNF-a, TL1A/DR3 axis, ovalbumin, asthma, EMT

## Abstract

Tumor necrosis factor (TNF)-like cytokine 1A (TL1A), a member of the TNF family, exists in the form of membrane-bound (mTL1A) and soluble protein (sTL1A). TL1A binding its only known functional receptor death domain receptor 3 (DR3) affects the transmission of various signals. This study first proposed that the TL1A/DR3 axis was significantly upregulated in patients and mice with both asthma and high TNF-a expression and in TNF-a-stimulated epithelial Beas-2B cells. Two independent approaches were used to demonstrate that the TL1A/DR3 axis of mice was strongly correlated with TNF-a in terms of exacerbating asthmatic epithelial–mesenchymal transformation (EMT). First, high expression levels of EMT proteins (e.g., collagen I, fibronectin, N-cadherin, and vimentin) and TL1A/DR3 axis were observed when mice airways were stimulated by recombinant mouse TNF-a protein. Moreover, EMT protein and TL1A/DR3 axis expression synchronously decreased after mice with OVA-induced asthma were treated with infliximab by neutralizing TNF-a activity. Furthermore, the OVA-induced EMT of asthmatic mice was remarkably improved upon the deletion of the TL1A/DR3 axis by knocking out the *TL1A* gene. TL1A siRNA remarkably intervened EMT formation induced by TNF-a in the Beas-2B cells. In addition, EMT was induced by the addition of high concentrations of recombinant human sTL1A with the cell medium. The TL1A overexpression *via* pc-mTL1A *in vitro* remarkably increased the EMT formation induced by TNF-a. Overall, these findings indicate that the TL1A/DR3 axis may have a therapeutic role for asthmatic with high TNF-a level.

## Introduction

Asthma, a heterogeneous disease, is characterized by chronic inflammation, epithelial–mesenchymal transformation (EMT), and airway remodeling (1). With the complexity of living environments, its inducing factors are becoming diverse and its incidence is increasing in young individuals, forcing the rapid rise of related medical costs and mortality worldwide (2, 3). Currently, most patients with asthma rely on drugs such as glucocorticoids and bronchodilators to improve disease progression. The underlying pathogenesis of asthma is particularly important to explore so that more effective and safer treatment options can be developed.

Chronic airway inflammation disorder exacerbates the progression of asthma (4). After immune system activation, macrophages, T cells, and other cells gather and secrete various factors, such as interleukin-1β, 4, 5, 10, and 13 and tumor necrosis factor (TNF)-a, which break the anti-inflammatory balance and aggravate asthma progression (5, 6). Interleukin (IL) is important for the initiation and transmission of allergic inflammation. Zhang et al. reported that IL-1β augments TGF-β-induced EMT of epithelial cells and is associated with poor pulmonary function in neutrophilic asthma (7). TNF-a, a member of the TNF superfamily, has promising applications in the pathophysiological progress of autoimmune diseases, such as Crohn’s disease, and the development and application of related drugs (8). Zhou et al. also revealed that TNF-a plays a critical role in the development of renal interstitial fibrosis (9). However, the mechanism in which TNF-a acts in mucus secretion, airway hyperreactivity, and airway remodeling of human asthma remains unclear.

TNF-like cytokine 1A (TL1A, a protein encoded by *TNFSF15*) is a type II transmembrane protein with a stable trimer structure similar to TNF-a (10). Migone et al. first uncovered the presence of TL1A as a membrane-bound protein (mTL1A) or a soluble protein (sTL1A) from mTL1A cleaved by an underlying enzyme (11). DR3 is a type I membrane protein that contains a death domain in the cytoplasmic region and remains highly homologous with other TNFRSF members. To date, the only confirmed TL1A receptor is DR3 (12, 13). The combination of TL1A with DR3 may play different or even opposite roles. TL1A/DR3 signaling regulates T cells to reduce inflammation while stimulating T effector cells to produce inflammatory cytokines (14). In addition, DR3 have multiple splicing variants, which may be the root cause of the diversity of physiological functions, such as DR3-mediated apoptosis (15), inflammatory/immune response (16), and herpes viral infection (17).

Interestingly, Bouros et al. found that TNF-a-stimulated human lung myofibroblasts remarkably increased TL1A expression and collagen production, but they did not further explore the mechanisms of TL1A in TNF-a-stimulated myofibroblasts (18). In the present study, the specific role of TL1A/DR3 axis in the EMT formation induced by TNF-a was identified. Moreover, blocking the TL1A/DR3 axis may be a novel therapeutic approach for asthma, because it inhibits the inflammatory response and improves EMT established by TNF-a.

## Materials and Methods

### Reagents

The recombinant proteins of human TNF-a (AF-300-01A) and mouse TNF-a (AF-315-01A) were obtained from PeproTech. The pcDNA3.1 and pc-mTL1A were constructed from FengHuiShengWu Biotechnology. Human TL1A siRNA (sc-39846) was obtained from Santa Cruz Biotechnology. TNF-a (ab215188) and collagen I (ab260043) antibodies were obtained from Abcam. Infliximab (A2019) was provided by Selleck Technology. Recombinant human sTL1A (1319-TL) was obtained from RD Systems. Antibodies of a-SMA (#19245), vimentin (#5741), and N-cadherin (#13116) were obtained from Cell Signaling Technology. Fibronectin (ET1702-25), GAPDH (ET1601-4), and HRP conjugated goat anti-rabbit IgG (HA1001) were from HUABIO. DR3 (#PA5-19882) was obtained from Invitrogen Technology. TL1A antibody (DF3053) was constructed by Affinity Biosciences. The HE staining kit (Cat#G1120), Masson staining kit (Cat#G1340), and broad spectrum SP kit (SP0041) were producted from SolarBio.

### Patient Samples

Healthy bronchial epithelial tissue samples were obtained from patients with suspected airway tumors, but no histopathological evidence of disease was found in these samples. Asthmatic samples were collected *via* bronchoscopy from patients diagnosed with asthma according to the 2019 Edition of the Global Asthma Initiative Criteria. The characteristics of human samples are shown in **Table 1**.

**Table 1:** Characteristics and physiologies of human samples.

Characteristic	Normal	Asthmatic
Number	6	7
Age (years)	57.8 ± 8.6	54.3 ± 14.0
Gender (male/female)	3/3	4/3
FEV1/FVC (% of predicted)	74.4 ± 5.5	51.9 ± 9.8
Eosinophils (10^9^)	0.10± 0.08	0.95± 0.77

Data are depicted as means ± SD. FEV1/FVC, the ratio of forced expiratory volume in the first second to forced vital capacity.

### Beas-2B Cell Treatment and Pre-Treatment

Beas-2B cells (Cell Line Bank, Shanghai) were derived from human bronchi and had epithelial-like appearance. Beas-2B cells were cultured in 6- or 24-well petri dishes (DMEM containing 10% FBS) to a density of 50%–70% and then used for all tests.

Beas-2B cells were stimulated in cell culture medium with TNF-a (50 ng/mL) for 12, 24, and 48 h to observe the effects on EMT and TL1A/DR3 axis. Then, we used 12.5, 25, and 50 ng/mL TNF-a for stimulation for 48 h in the Beas-2B cells.

Different concentrations of recombinant human sTL1A protein in the Beas-2B cell culture were stimulated for 48 h to observe the effects of exogenous sTL1A on EMT. In addition, cells were assigned to the control, NC siRNA, TNF-a, and TL1A siRNA+TNF-a groups. Cells in the NC siRNA group were transfected with NC siRNA by Lipofectamine 3000 for the negative control. Cells in the TNF-a group were cultured in cell culture medium with TNF-a (50 ng/mL) stimulation 48 h. Cells in the TL1A siRNA+TNF-a group were transfected with 20 nM TL1A siRNA to intervene TL1A for 12 h, and then stimulated with 50 ng/mL TNF-a to observe the effect of TL1A intervention on TNF-a-induced EMT.

TL1A overexpression was established by pc-mTL1A to observe the effects on the TNF-a-stimulated Beas-2B cells. Cells in the control group were harvested when the density reached 50%–70% without any treatment. Cells in the pcDNA3.1 group were transfected with pcDNA3.1 by using Lipofectamine 3000 for the negative control. Cells in the pc-mTL1A group were transfected with pc-mTL1A by using Lipofectamine 3000 to overexpress mTL1A. Cells in the TNF-a group were cultured in cell culture medium by stimulating with 50 ng/mL TNF-a for 48 h. Cells in the pc-mTL1A+TNF-a group were transfected with pc-mTL1A by using Lipofectamine 3000 according to the manufacturer’s protocol, and then cells in the cell culture medium were stimulated with 50 ng/mL TNF-a for 48 h.

### Mice and Grouping

*TL1A^+/-^* female and male mice (18–20 g) were obtained from Nanjing University Institute of Zoology, and then bred and raised in the Qianfoshan Hospital at SPF environment. Female and male *TL1A^+/-^* mice were mated, and the pups of *TL1A^-/-^* and *TL1A^+/+^* females were selected *via* genetic identification. According to the international asthma animal model, female mice were selected for asthma study (19). Female and male *TL1A^+/-^* pups were used for reproduction and breeding. Mice were studied in accordance with the National Institutes of Health Guidelines for the Care and Use of Laboratory Animals and approved by the Qianfoshan Hospital Institutional Review Committee.

**Figure 1:**
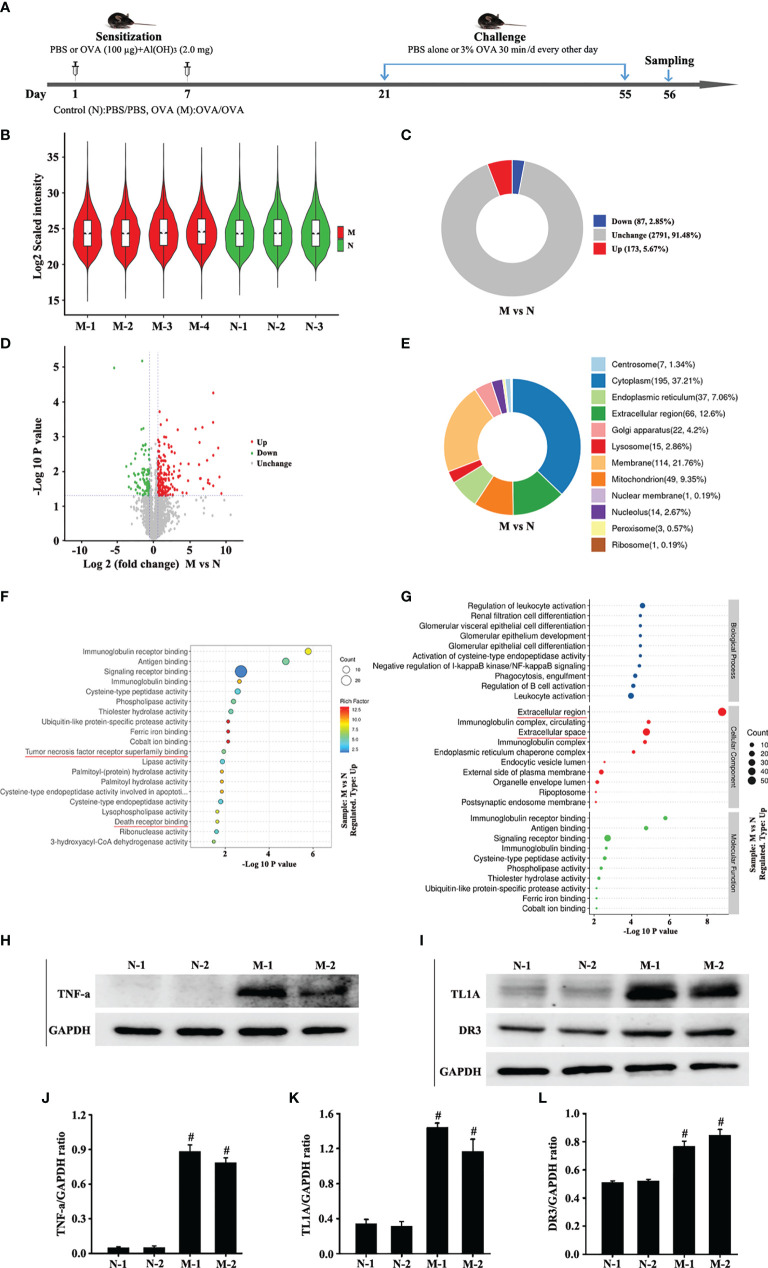
Animal experimental schedule is shown in **(A)** (*n* = 6 in each group). Analysis of differentially expressed proteins (DEPs) in OVA-induced asthma (M) compared with control (N). The boxplot shows the distribution of sample expression **(B)**. The pie chart shows the number and proportion of DEPs between groups **(C)**. The volcano plot exhibits the quantitative expression of DEPs between groups **(D)**. The pie chart displays the subcellular localization of DEPs between groups **(E)**. The chart shows the term of the top 20 significance of differential protein enrichment between groups **(F)**. The chart illustrates the biological process, cellular component, and molecular function **(G)**. Top 10 term of difference protein enrichment in the three branches **(G)**. Finally, we combined the differential protein dataset to detect the changes of TNF-a **(H)** and TL1A/DR3 **(I)** in the mouse lungs *via* Western blotting analysis. **(J)** intensity analysis of **(H)**. **(K, L)** intensity analysis of **(I)**. Data are expressed as the means ± SD for three independent experiments. ^#^p < 0.05 versus the control group.

Wild female mice were randomly divided into the control and OVA groups to observe the changes in TNF-a and TL1A/DR3 axis (*n* = 6 per group). The effects of TNF-a on the TL1A/DR3 axis and airway EMT in mice were assessed using two independent methods. First, the airways of wild female mice were stimulated with mouse TNF-a protein. Second, wild female mice were divided into the control, infliximab, OVA, and OVA+infliximab groups. The preparation of mouse model is illustrated in **Figures 1A**, **3A**, **4A**.

Finally, mice were divided into the control ^TL1A+/+^, control ^TL1A-/-^, OVA ^TL1A+/+^, and OVA ^TL1A-/-^ groups to evaluate the effect of target knockout *TL1A* gene on asthma. The process of mouse model preparation is shown in **Figure 5A**.

### Label-Free Quantitative Proteomics Analysis

The control (N) group and OVA-induced asthma (M) group consisted of three and four mouse samples, respectively. Label-free 10-standard reagent was used for labeling and quantification. An appropriate amount of lung tissues was added with 200 µL of SDT lysate for ultrasonic crushing in ice bath. Lung supernatant was obtained after centrifugation at 4 °C and 16,000 ×g. Lung protein samples (20 µg) were obtained from each group at 5:1 (V/V), added into the 5× loading buffer solution, and then bathed in boiling water for 5 min. SDS-PAGE gel electrophoresis and coomassie blue staining were performed. Lung protein samples (300 µg) were obtained from each sample for enzymatic hydrolysis. A proper amount of 1 M DTT was added to each sample to a final concentration of 10 mM. Then, the sample was bathed in boiling water for 5 min and cooled to room temperature. An appropriate amount of 800 mM IAA was added to a final concentration of 50 mM. The solution was shaken at 600 rpm for 1 min and kept away from light for 30 min at room temperature. Add 6 times the volume of cold acetone reagent, and the solution was incubated overnight at −20 °C to precipitate the protein. The solution was centrifuged at 16,000 ×g for 30 min, the supernatant was removed, and the protein precipitate was retained. An appropriate amount of acetone reagent was added to clean the protein precipitate, and the process was repeated twice. The protein precipitated sample was dried in a fume hood to remove the organic reagent. Approximately 150 µL of trypsin buffer (6 µg trypsin in 144 µL NH_4_HCO_3_ buffer) was added, oscillated at 600 rpm for 1 min, incubated at 37 °C for 16–18 h, and then subjected to enzymatic hydrolysis. After adding 0.1% TFA solution, the peptide was desalted using C_18_ cartridge and freeze-dried in vacuum. After enzymatic hydrolysis, the peptide was dried and redissolved with 0.1% TFA, and then desalted in the thermo desalting spin column to quantify the peptide. An appropriate amount of peptide was obtained from each sample for chromatographic separation by using an EASY-nLC 1200 chromatography system (Thermo Scientific). The obtained LC-MS/MS RAW files were finally imported into the search engine sequest HT in proteome discoverer software (version 2.4, Thermo Scientific) for database retrieval. The above lung tissue samples were tested by Shanghai Bioprofile Technology Company Ltd.

### Mouse Airway Resistance Assessment

The whole-body plethysmography (WBP) system was located in a separate room to keep the surrounding environment quiet. The mice were placed in the test box and allowed to adapt to the environment for 30 min. The airway resistance indexes including Penh were measured using the WBP system with gradient concentrations of acetylcholine stimulation (20).

### Mouse Airway Injury Assessment

Mice were added with 4% paraformaldehyde to fix the lung tissue through pulmonary circulation. The lung tissue was cut into blocks with dimensions of 0.3 cm×0.3 cm×0.5 cm, embedded in paraffin, and cut into 5 µm-thick sections. Hematoxylin-eosin staining (HE) was used to assess the degree of airway inflammation (21). Collagen deposition around the airway was measured by Masson staining (21).

### Immunohistochemistry

Lung tissue sections were immersed in citric acid repair solution to perform antigenic repair under high temperature and pressure. After natural cooling, lung tissue sections were incubated with serum and antibody (TL1A, 1:200; DR3, 1:150; vimentin, 1:200; N-cadherin, 1:100) for 12 h at 4 °C. The next day, the lung tissues were washed thrice with PBS (5 min/time), and the goat anti-rabbit antibody (1:200) was incubated in a dark room for 30 min. Finally, the lung tissue sections were stained with DAB and hematoxylin, followed by dehydration and transparency. Finally, the protein expression was evaluated by microscopy and Image J analysis.

### Western Blotting Analysis

The concentration of different protein samples was detected using BCA protein assay kit. The protein samples in 10% separation gel were separated by electrophoresis at 80 V for 30 min and 120 V for 70 min, and then transferred to 0.45 or 0.2 µm PVDF membranes. Antibodies (TNF-a, 1:3,500; collagen I, 1:4,000; TL1A, 1:2,000; DR3, 1:3,000; vimentin, 1:2,500; N-cadherin, 1:2,500; fibronectin, 1:1,000; GAPDH, 1:5,000) were incubated at room temperature for 7 h after PVDF membrane infiltration with 3% BSA for 70 min. The PVDF membrane was washed with TBST solution, and the second antibody (1:5,000) was incubated for 1.5 h. Finally, TBST was used to clean the PVDF membranes, and chemiluminescence reagent was used to observe changes in PVDF membrane protein expression.

### Immunofluorescence

Lung tissue sections were dewaxed, dehydrated, and treated with citric acid repair solution for antigenic thermal repair. After natural cooling and PBS washing, the lung tissue sections were sealed with goat serum for 30 min. The first antibody (a-SMA, 1:200; TNF-a, 1:200; collagen I, 1:150; DR3, 1:150; vimentin, 1:200; N-cadherin, 1:100) was incubated on the surface of lung tissue sections for 12 h at 4 °C. Finally, the lung tissue sections were washed by PBS, incubated with fluorescence secondary antibody (dark chamber, 1:200) and DAPI (dark chamber, 1:200), and photographed under a fluorescence microscope.

Slides were placed in the 24-well plate under aseptic conditions, in which Beas-2B cells were evenly planted. Beas-2B cells were treated with 4% paraformaldehyde for 30 min. After washing with PBS, Beas-2B cells were incubated with the first antibody (TL1A, 1:200; collagen I, 1:150; vimentin, 1:200; fibronectin, 1:200; N-cadherin, 1:100) at 4 °C for at least 12 h. Beas-2B cells were washed with PBS, incubated with fluorescent secondary antibodies (dark chamber, 1:200) and DAPI (dark chamber, 1:200), and photographed under a fluorescence microscope.

### Statistical Analysis

Data are presented as mean ± SEM. Two-tailed and unpaired t-tests were used for comparison between two groups. One-way ANOVA was performed for comparison among multiple groups. P < 0.05 was considered statistically significant.

## Results

### TL1A/DR3 Axis Is Overexpressed in the Asthmatic Mice With High TNF-a Expression Induced by OVA

The Log2 scaled intensity was close to the same horizontal line, indicating good data quality and repeatability (**Figure 1B**). The criteria for screening differentially expressed proteins (DEPs) are p < 0.05 and cut-off fold change >1.5. The number and subcellular localization of DEPs were exhibited (**Figures 1C–E**). The chart showed the top 20 significant differences in protein enrichment between groups, including TNF receptor superfamily and death receptor binding protein (**Figure 1F**). The top 10 protein enrichment differences in biological processes, cell composition, and molecular function were shown in **Figure 1G**. These results suggest that TNF receptor superfamily and death receptor might be related to remodeling or EMT. Finally, this study combined the differential protein dataset to detect the changes of TNF-a and TL1A/DR3 in animal models. Western blotting analysis results suggest that the expression of TNF-a and TL1A/DR3 remarkably increased in OVA-induced asthma model compared with control group (**Figures 1H–L**).

### TL1A/DR3 Axis Is Overexpressed in the Asthmatic Bronchial Epithelium of Patients With High TNF-a Expression

**Figure 2:**
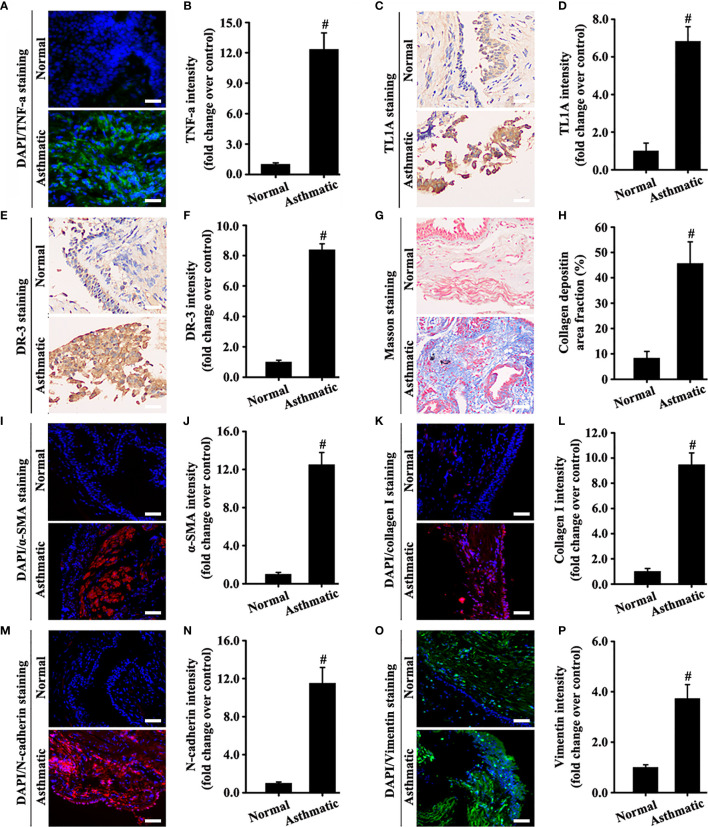
Human bronchial epithelium of asthmatic and normal samples was detected using different experimental techniques. The levels of TNF-a (**A**, green), α-SMA (**I**, red), collagen I (**K**, red), N-cadherin (**M**, red), and vimentin (**O**, green) were measured by immunofluorescence. The levels of TL1A (**C**, pale brown) and DR3 (**E**, pale brown) were evaluated by immunohistochemistry. Collagen deposition was determined by Masson staining (**G**, blue). The blue color **(A, I, K, M, O)** represents DAPI. **(A, C, E, G, I, K, M, O)**: magnification 200 ×, scale bar 50 µm. **(B, D, F, H, J, L, N, P)** intensity analysis of **(A, C, E, G, I, K, M, O)**. Data are expressed as the means ± SD for three independent experiments. ^#^p < 0.05 versus the normal group.

Results of specimens from patients with asthma showed that TNF-a and the TL1A/DR3 axis were overexpressed simultaneously in the airway epithelium (**Figures 2A–F**). Masson staining and immunofluorescence staining of the EMT index (a-SMA, collagen I, N-cadherin, and vimentin) showed a large amount of collagen deposition around the airway of patients with asthma and obvious mesenchymal transformation of epithelial cells (**Figures 2G–P**). In combination with the results in **Figures 1**, **2**, we hypothesized that the TL1A/DR3 axis might be involved in EMT mediated by TNF-a.

### Intratracheal Atomization of TNF-a Aggravates the TL1A/DR3 Axis and EMT in the Lungs of Mice

**Figure 3:**
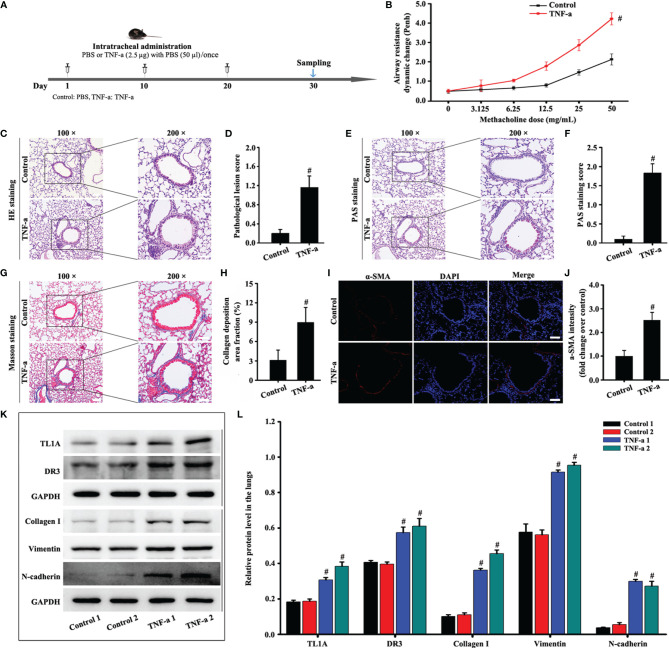
Animal experimental schedule is shown in **(A)** (*n*= 6 in each group). Effects of recombinant mouse TNF-a protein on mice. Airway resistance was detected using the WBP system **(B)**. HE **(C)**, PAS **(E)**, Masson **(G)**, and a-SMA staining **(I)** were evaluated in animal models. Effects of TNF-a on TL1A, DR3, collagen I, fibronectin, vimentin, and GAPDH in the mice were detected by Western blotting analysis **(K)**. **(C, E, G)**: magnification 100 ×, scale bar 100 µm; magnification 200 ×, scale bar 50 µm. **(I)**: magnification 200 ×, scale bar 50 µm. **(D)** bronchial inflammation score of **(C)**. **(F)** mucus intensity of **(E)**. **(H)** collagen deposition intensity of **(G)**. **(J, L)** protein intensity analysis of **(I, K)**. mData are expressed as the means ± SD for three independent experiments. ^#^p < 0.05 versus the control group.

TNF-a expression remarkably increased in the asthma model compared with the control. Therefore, we stimulated the recombinant mouse TNF-a protein for 30 days to observe the effect on the TL1A/DR3 axis and EMT. The WBP system suggested that the recombinant TNF-a protein aggravated airway resistance in mice (**Figure 3B**). In addition, recombinant TNF-a protein remarkably increased the accumulation of airway inflammatory cells and airway epithelium mucus secretion in mice (**Figures 3C–F**). Interestingly, mice stimulated with the mouse recombinant TNF-a protein also increased the TL1A/DR3 axis and EMT (**Figures 3G–L**). Therefore, TNF-a may influence EMT *via* the TL1A/DR3 axis.

### TL1A/DR3 Axis and EMT Induced by OVA Synchronously Decrease After Infliximab by Specifically Neutralizing TNF-a Activity

**Figure 4:**
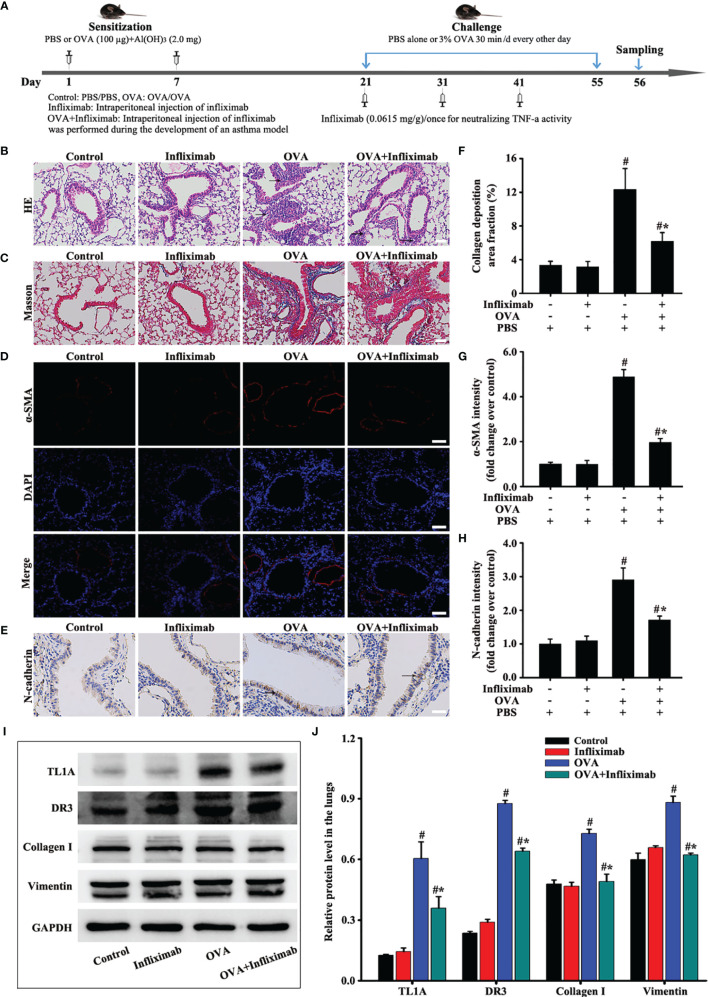
Animal experimental schedule is shown in **(A)** (*n*= 6 in each group). Effects of infliximab on OVA-induced asthma were observed. HE **(B)**, Masson **(C)**, a-SMA **(D)**, and N-cadherin staining **(E)** were detected. The effects of infliximab on TL1A, DR3, collagen I, and vimentin were detected by Western blotting analysis **(I)**. **(B, C)**: magnification 100 ×, scale bar 100 µm. **(D, E)**: magnification 200 ×, scale bar 50 µm. **(F–H, J)** intensity analysis of **(C–E, I)**. Data are expressed as the means ± SD for three independent experiments. ^#^p < 0.05 versus the control group. ^#^*p < 0.05 versus the OVA group.

The specific neutralization of TNF-a by infliximab on the TL1A/DR3 axis and EMT was observed in mice with OVA-induced asthma. Therefore, infliximab improved the accumulation of airway inflammatory cells and airway remodeling in mice induced by OVA (**Figures 4B, C, F** **)**. Interestingly, the TL1A/DR3 axis and EMT were remarkably improved after infliximab specifically neutralized OVA-induced TNF-a activity in asthmatic mice (**Figures 4D, E, G–J**). These results confirm that TNF-a influenced EMT formation through the TL1A/DR3 axis.

### Knockout *TL1A* Decreases OVA-Induced EMT in Mice

**Figure 5:**
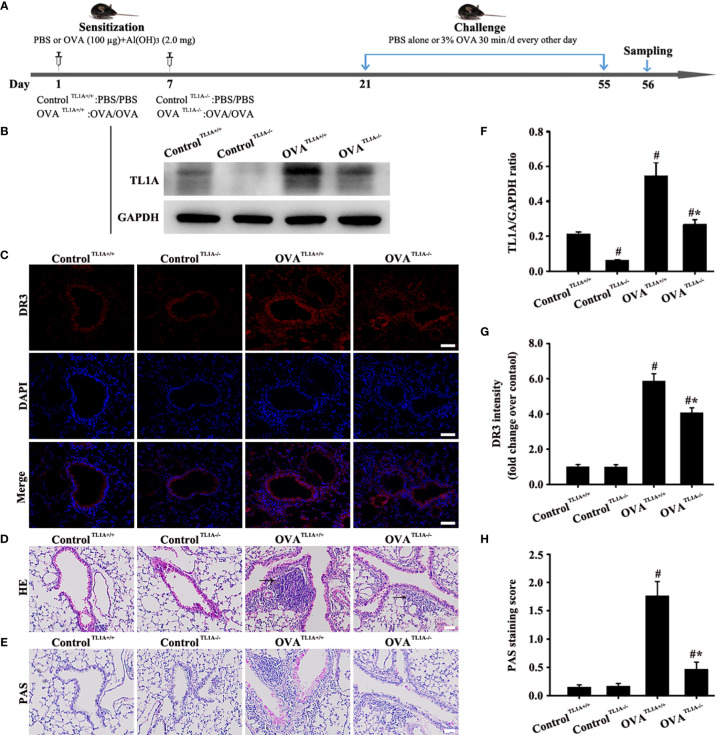
Animal experimental schedule is shown in **(A)** (*n*= 6 in each group). Effects of the knockout *TL1A* gene on OVA-induced asthma were observed. TL1A levels in the lung as determined by Western blot analysis **(B)**. Effects of the knockout *TL1A* gene on DR3 **(C)**, HE **(D)**, PAS staining **(E)** were detected. **(C)**: magnification 200 ×, scale bar 50 µm. **(D, E)**: magnification 100 ×, scale bar 100 µm. **(F–H)** intensity analysis of **(B, C, E)**. Data are expressed as the means ± SD for three independent experiments. ^#^p < 0.05 versus the control group. ^#^*p < 0.05 versus the OVA group.

**Figure 6:**
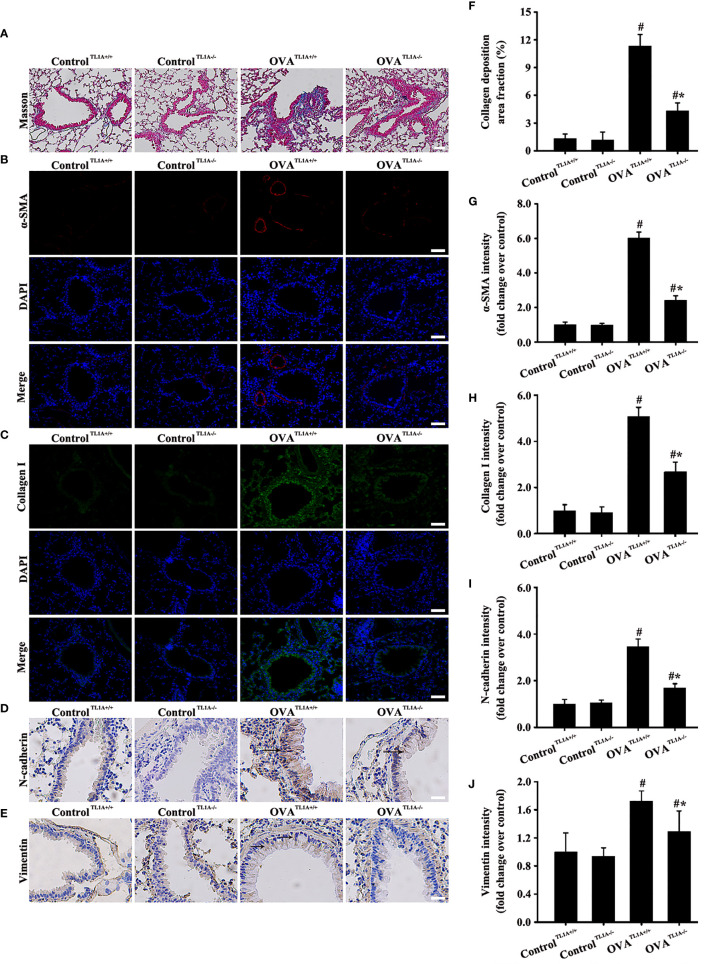
Animal experimental schedule is shown in **Figure 5A** (*n*= 6 in each group). Effects of the knockout *TL1A* gene on the EMT in mice were observed. The effects of the knockout *TL1A* gene on Masson **(A)**, a-SMA **(B)**, collagen I **(C)**, N-cadherin staining **(D)**, and vimentin staining **(E)** were detected. **(A)**: magnification 100 ×, scale bar 100 µm. **(B–E)**: magnification 200 ×, scale bar 50 µm. **(F–J)** intensity analysis of **(A–E)**. Data are expressed as the means ± SD for three independent experiments. ^#^p < 0.05 versus the control group. ^#^*p < 0.05 versus the OVA group.

TL1A expression in lung tissue remarkably decreased in OVA-induced asthmatic mice after the deletion of the *TL1A* gene in mice (**Figures 5B, F**). Immunofluorescence result of DR3 suggested that *TL1A* knockout affected DR3 expression in asthmatic mice (**Figures 5C, G**). HE and PAS results showed that *TL1A* knockout improved the airway inflammation and mucus secretion in OVA-induced mice (**Figures 5D, E, H**). Notably, TL1A deletion remarkably improved airway remodeling and EMT in OVA-induced mice (**Figures 6A–J**). Therefore, the TL1A/DR3 axis was involved in EMT formation in asthmatic mice.

### TL1A/DR3 Axis and EMT in Beas-2B Cells Are Overexpressed by TNF-a Stimulation

**Figure 7:**
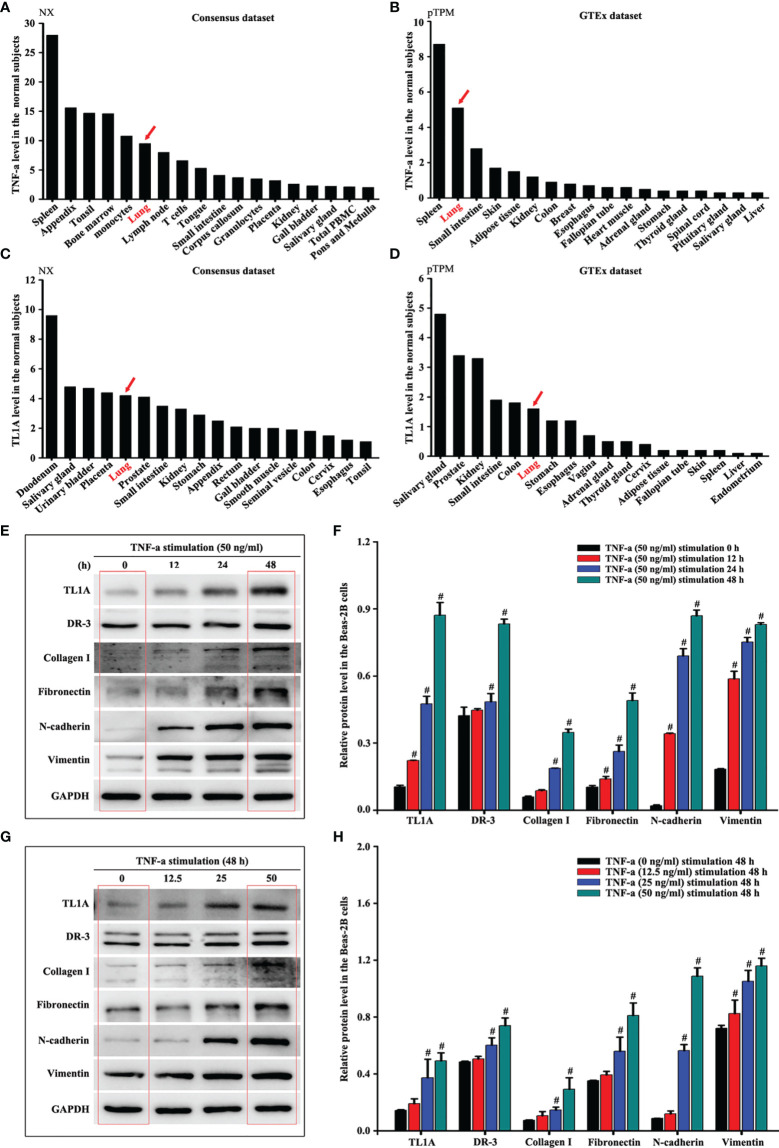
Human Tissue Atlas (http://www.proteinatlas.org/) was used to identify the protein levels of TNF-a **(A, B)** and TL1A **(C, D)**. The same concentration of TNF-a stimulated Beas-2B cells at different times **(E)** or different concentrations of TNF-a stimulated Beas-2B cells at the same time **(G)** to evaluate the effects of TNF-a on TL1A, DR3, collagen I, fibronectin, vimentin, and GAPDH by Western blotting analysis. **(F, H)** intensity analysis of **(E, G)**. Data are shown as the means ± SD for three independent experiments. ^#^p < 0.05 versus the control group.

Human Tissue Atlas (http://www.proteinatlas.org/) was used to identify the protein levels of TL1A and TNF-a which were higher in normal lungs than in most other organs (**Figures 7A–D**). Considering the importance of airway epithelial cells in asthma, we then selected TNF-a to stimulate Beas-2B cells and thus observe the effect of the TL1A/DR3 axis and EMT. The results showed that the TL1A/DR3 axis and EMT (collagen I, fibronectin, N-cadherin, and vimentin) remarkably changed at 48 h with TNF-a stimulation time (**Figures 7E, F**). We selected different concentrations of TNF-a for stimulation for 48 h. Results show remarkable changes in the TL1A/DR3 axis and EMT at 50 ng/mL with the increase in TNF-a stimulation concentration (**Figures 7G, H**). Accordingly, we selected 50 ng/mL TNF-a stimulation for 48 h as the best cell model.

### TL1A/DR3 Axis, A Key Target of TNF-a, Augments EMT

**Figure 8:**
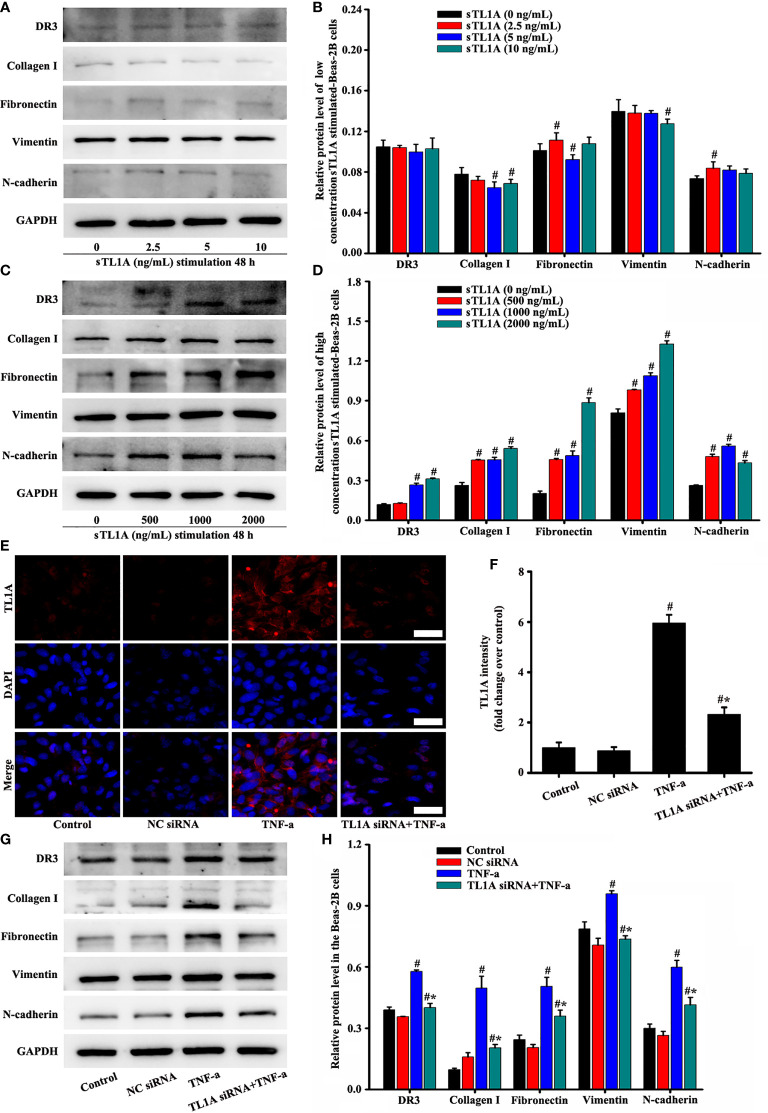
Effects of the TL1A/DR3 axis on the EMT in Beas-2B cells were detected. TNF-a (50 ng/mL) stimulation for 48 h as the best cell model based on the above experimental results. The effect of sTL1A on EMT was detected by Western blotting **(A, C)**. The effects of TL1A siRNA on TL1A induced by TNF-a were detected by immunofluorescence **(E)**. Effects of TL1A siRNA on the EMT formation induced by TNF-a were measured by Western blotting analysis **(G)**. **(E)**: magnification 200 ×, scale bar 50 µm. **(B, D, F, H)** intensity analysis of **(A, C, E, G),** respectively. Data are expressed as the means ± SD for three independent experiments. ^#^p < 0.05 versus the control group. ^#^*p < 0.05 versus the TNF-a group.

Based on the above results, we further explored the role of the TL1A/DR3 axis in EMT-induced TNF-a. Subsequently, we added different concentrations of sTL1A into the cell culture medium to verify the effect of sTLA. DR3 and most EMT indexes did not remarkably increase after the addition of low concentration of sTLA (0–10 ng/mL) in external cell culture (**Figures 8A, B**). In addition, the concentration of sTL1A (0, 200, 400 ng/ml) did not remarkably induce the EMT indexes of epithelial cells. Considering the effect of COVID-19 pandemic, these data (the sTL1A concentration of 0, 200, 400 ng/ml) have not been edited and presented. After the addition of 500–2,000 ng/mL sTLA to the external cell culture, DR3 and EMT remarkably increased (**Figures 8C, D**). DR3 levels were reduced, and EMT transformation was improved after the intervention of TL1A siRNA in TNF-a-induced TL1A (**Figures 8E–H**).

**Figure 9:**
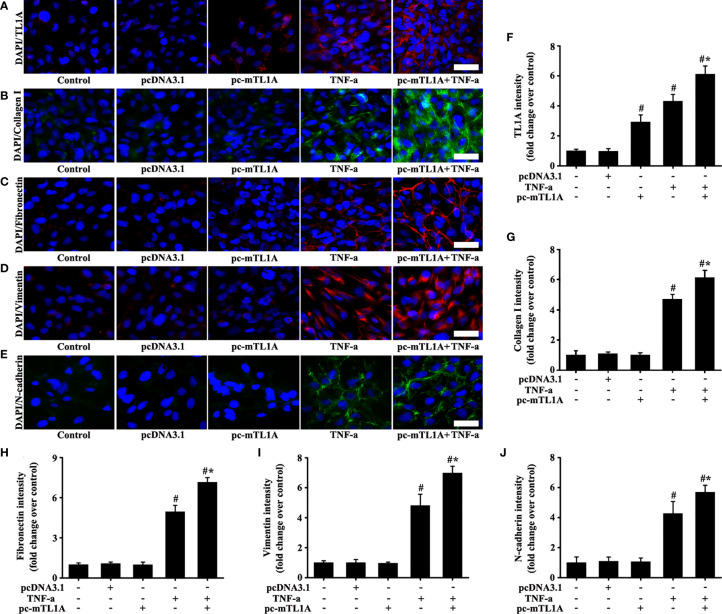
Effects of TL1A overexpression by pc-mTL1A on the EMT formation in Beas-2B cells. TNF-a (50 ng/mL) stimulation for 48 h as the best cell model based on the experimental results. The levels of TL1A (**A**, red), collagen I (**B**, green), fibronectin (**C**, red), vimentin (**D**, red), and N-cadherin (**E**, green) were detected by immunofluorescence. **(A–E)**: magnification 200 ×, scale bar 50 µm. **(F–J)** intensity analysis of **(A–E)**, respectively. Data are expressed as the means ± SD for three independent experiments. ^#^p < 0.05 versus the control group. ^#^*p < 0.05 versus the TNF-a group.

Immunofluorescence results showed that TL1A overexpression was effective in pc-mTL1A+TNF-a group (**Figures 9A, F**). The expression levels of collagen I, fibronectin, vimentin, and N-cadherin increased to a certain extent in the pc-mTL1A+TNF-a group compared with those in the TNF-a group (**Figures 9B–E, G–J**).

Overall, TL1A/DR3 axis plays a key role in enhancing EMT formation induced by TNF-a.

## Discussion

Chronic inflammatory response accompanied with EMT is an important feature in the development of asthma (22). Epithelial cells evolve from barrier functions to binding targets, thus aggravating disease signals (23). This study was the first to demonstrate that the TL1A/DR3 axis is actively involved in EMT stimulated by TNF-a at the cellular, mouse, and human levels.

TNF-a, a trimer structure, is produced by membrane anchoring precursor proteins such as macrophages, monocytes, and T cells, which are subsequently cleaved by TNF-a conversion enzymes into free and active TNF-a protein (24). TNF-a binds to cell surfaces with TNF-a receptors during mobile dissociation and participates in inflammatory and immune signaling (25, 26). TNF-a and TL1A are both members of the TNF superfamily. Migone et al. reported that TL1A expression is induced by TNF and IL-1a in HUVEC cells (11). Jin et al. found that TL1A induces proinflammatory cytokine TNF-a from isolated human CD4+CD161+ T cells (27). Our study found that TL1A expression was induced by TNF-a in Beas-2B cells and was involved in EMT formation. Therefore, a positive feedback loop is present between the two cytokines. Previous study results in the RA patient serum have revealed the inhibition of TL1A production *via* anti-TNF-a (28). The results of the present study supports these findings, in which the inhalation of TNF-a induce TL1A/DR3 expression in mice, and the reduction of TL1A/DR3 in asthmatic mice is induced by OVA after anti-TNF-a treatment.

TL1A in the form of mTL1A and sTL1A has only one confirmed receptor, namely, DR3. Migone et al. reported that the sTL1A from mTL1A is cleaved by an underlying enzyme (11). The ligand TL1A binds to the receptor DR3 to promote lymphocyte co-stimulation, mucosal hyperplasia, and autoimmune inflammation (29–31). Herro et al. found that TL1A promotes lung tissue fibrosis (32). Steele et al. also showed that targeting TL1A/DR3 signaling offers a therapeutic advantage in neutralizing IL13/IL4Ralpha in muco-secretory fibrotic disorders (33). TL1A maintains the blood–retinal barrier by modulating SHP-1-Src-VE-cadherin signaling in the diabetic retinopathy (34). Notably, the present study found that TNF-a was closely associated with TL1A/DR3 in asthma, and the TL1A/DR3 targets play a key role in EMT induced by TNF-a. Interestingly, the data uncovered that TL1A overexpression by pc-mTL1A alone did not induce EMT mean TL1A signaling alone was not enough to induce EMT, while it was indispensable for the action of TNF-a. The data might be attributed to the fact that TL1A overexpression by pc-mTL1A alone did not reach the threshold to induce changes in DR3 receptor. However, TL1A overexpression in TNF-a+ pc-mTL1A group might enable TL1A to obtain more opportunities to bind DR3 receptor, and then transmitted TL1A/DR3 signaling. These results indicated that TL1A/DR3 axis was a key point in TNF-a-induced EMT formation. These results also explain how TNF-a increases the airway hyper-responsiveness in both normal subjects and patients with asthma.

The imbalance in inflammatory response may be one of the important causes of EMT in chronic asthma (35). Pro-inflammatory cytokines include IL-1β, 2, 6, 8, 12, 17, LT-α, TNF-a, and IFN-γ. The typical anti-inflammatory cytokine interleukin-10 (IL-10) is the inhibitor of human cytokine synthesis. IL-10 signaling is transmitted *via* two IL-10 homologous dimers with a hetero-tetramer receptor (36, 37). The recombinant human IL-10 protein could remarkably inhibit the expression of TL1A/DR3 axis and EMT formation in the Beas-2B cells stimulated by TNF-a. The same phenomena were observed after injection with recombinant mouse IL-10 protein into the asthmatic mice (this part of the result is not shown). Therefore, IL-10 may affect the formation of asthmatic EMT through TL1A/DR3 targets mediated by TNF-a, thus further enriching the signaling pathway of IL-10.

ATF3, a key regulator, belongs to the CAMP response element binding protein family with basic leucine zipper structure (BZIP) (38). ATF3 plays different or even opposite roles in different cellular states by transforming into an isodimer or by combining with other BZIP proteins, which is involved in pathological processes, such as oxidative stress, apoptosis, regulation of Th2 cytokines, and signal transduction (39, 40). Gilchrist et al. reported that ATF3 is a negative regulator of allergic pulmonary inflammation (41). TL1A/DR3 and EMT-related index also increased in asthmatic mice after ATF3 intervention (this part of the result is not shown). In addition, TNF-a induced ATF3 expression in Beas-2B cells (this part of the result is not shown). Subsequently, ATF3 regulatory TL1A/DR3 signaling was enriched in OVA-induced asthma.

Although this study supports the conclusions to a certain extent, some limitations were observed. The higher concentration of sTL1A required to induce EMT might be due to various possibilities, and one of them is the much weaker bioactivity of commercial recombinant protein. In addition, *TL1A* knockout mice were constructed *in vivo* to explore the role of TL1A/DR3 in asthma. However, *DR3* knockout mice has not been constructed because of financial and technical reasons.

In conclusion, this study is the first to demonstrate that TNF-a may regulate EMT in epithelial cells through the TL1A/DR3 signaling pathway. This study also provides a new theoretical basis and ideas for the treatment of asthma in the future.

## Data Availability Statement

The original contributions presented in the study have been uploaded to the ProteomeXchange Consortium *via* the PRIDE partner repository with the dataset identified PXD031079. Further inquiries can be directed to the corresponding author.

## Ethics Statement

The studies involving human participants were reviewed and approved by Qianfoshan Hospital Institutional Review Committee. The patients/participants provided their written informed consent to participate in this study. The animal study was reviewed and approved by Qianfoshan Hospital Institutional Review Committee. Written informed consent was obtained from the individual(s) for the publication of any potentially identifiable images or data included in this article.

## Author Contributions

DZ conducted the study, performed part of the animal and cell operations, drafted the figures, and wrote the original draft. HY performed part animal operations, wrote and proofread the original draft. X-LD made some contributions to the manuscript revision work during the COVID-19 pandemic. J-TZ performed part of the animal operation and drafted some figures. JZ was provided human bronchial epithelial samples. W-JC interpreted the data and drafted the figures. YP, X-FL, and J-WX fed the Beas-2B cells, drafted some figures, and revised the manuscript. Z-HW provided big data support. LD conceived the idea, provided experimental funds, and performed rigorous revision. All authors approved the submitted version of the article.

## Funding

The present study work was supported by the National Natural Science Foundation of China (81770029), Key Research and Development Program of Shandong Province (2021SFGC0504), and Shandong Provincial Natural Science Foundation (ZR2021LSW015).

## Conflict of Interest

The authors declare that the research was conducted in the absence of any commercial or financial relationships that could be construed as a potential conflict of interest.

## Publisher’s Note

All claims expressed in this article are solely those of the authors and do not necessarily represent those of their affiliated organizations, or those of the publisher, the editors and the reviewers. Any product that may be evaluated in this article, or claim that may be made by its manufacturer, is not guaranteed or endorsed by the publisher.
